# The Role of Executive Function in the Effectiveness of Multi-Component Interventions Targeting Physical Activity Behavior in Office Workers

**DOI:** 10.3390/ijerph19010266

**Published:** 2021-12-27

**Authors:** Rui Wang, Victoria Blom, Carla F. J. Nooijen, Lena V. Kallings, Örjan Ekblom, Maria M. Ekblom

**Affiliations:** 1 Department of Physical Activity and Health, the Swedish School of Sport and Health Sciences, GIH, 11433 Stockholm, Sweden; victoria.blom@gih.se (V.B.); Carla.Nooijen@permobil.com (C.F.J.N.); lena.kallings@gih.se (L.V.K.); orjan.ekblom@gih.se (Ö.E.); maria.ekblom@gih.se (M.M.E.); 2 Division of Clinical Geriatrics, Department of Neurobiology, Care Sciences and Society, Karolinska University Hospital at Huddinge, 14157 Huddinge, Sweden; 3 Wisconsin Alzheimer’s Disease Research Center, University of Wisconsin School of Medicine and Public Health, Madison, WI 53726, USA; 4 Department of Psychology, Stockholm University, 10691 Stockholm, Sweden; 5 Department of Public Health and Caring Sciences, Family Medicine and Preventive Medicine, Uppsala University, 75236 Uppsala, Sweden; 6 The Department of Neuroscience, Karolinska Institutet, 17176 Stockholm, Sweden

**Keywords:** physical activity, sedentary behavior, executive function, job control, job demands, active jobs, self-regulation, health promotion

## Abstract

A knowledge gap remains in understanding how to improve the intervention effectiveness in office workers targeting physically active (PA) behavior. We aim to identify the modifying effect of executive function (EF) on the intervention effectiveness targeting PA-behaviors, and to verify whether the observed effect varies by Job Demand Control (JDC) categories. This workplace-based intervention study included 245 participants who were randomized into a control group and two intervention arms—promoting physical activity (iPA) group or reducing sedentary behavior (iSED) group. The interventions were conducted through counselling-based cognitive behavioral therapy and team activities over 6 months. PA-behaviors were measured by an accelerometer. EF was assessed by the Trail Making Test-B, Stroop, and n-back test. The JDC categories were measured by the demand control questionnaire. Higher EF level at baseline was significantly associated with the intervention effect on increased sleep time (β-coefficient: 3.33, *p* = 0.003) and decreased sedentary time (−2.76, *p* = 0.049) in the iSED-group. Participants with active jobs (high job demands, high control) presented significantly increased light-intensity PA in the iSED-group in comparison to the control group. Among participants with a high level of EF and active jobs, relative to the control group, the iPA-group showed a substantial increase in light-intensity PA (1.58, *p* = 0.036) and the iSED-group showed a tendency of reducing sedentary behavior (−5.35, *p* = 0.054). The findings suggest that office workers with a high EF and active jobs may benefit most from an intervention study targeting PA-behaviors.

## 1. Introduction

A habitually physically active lifestyle can reduce all-cause mortality and improve several health outcomes [1]. Yet, a knowledge gap remains in understanding how to motivate people to become more physically active or to be less sedentary, especially among employed populations with long-term sedentary work [2]. Self-regulation theory has suggested that the relation between PA-related behavior and cognitive control is reciprocal. That is, executive function (EF) plays an antecedent role in the effective self-regulation of PA behavior [3]. A bidirectional relationship between PA and EF has been observed in the population with a wide range of ages [4,5,6]. PA can promote the development and preservation of EF in early life, and therefore benefit the self-regulation process of PA-behaviors in adulthood [7]. The EF, on the other hand, enables individuals to engage in healthy behaviors, such as reducing sedentary behavior and quitting smoking [3]. Therefore, studying the role of EF in PA-behaviors is critical for understanding the effectiveness of PA-related interventions.

Findings remain inconsistent in studies that employed single- or multi-component interventions targeting PA and sedentary behavior, particularly in office workers or workplace-based studies [7,8,9,10,11,12,13]. Some studies have found that workplace-based interventions on PA or sedentary behavior were effective in promoting PA, reducing sitting time, breaking prolonged sitting periods, or improving general health [7,9,10,11,13], while other studies did not observe any significant intervention effect on either increasing PA or reducing sedentary time among office workers [8,12]. Although the inconsistent results can be attributable to the diversity in the length of interventions, sizes of enterprises, and occupational populations, it is encouraged to identify the modifiable factors that can influence the effectiveness of PA-related interventions [1]. To our best knowledge, no study has previously investigated the link between self-regulation related cognitive factor (i.e., EF) and effectiveness of PA-related interventions in a workplace-based setting.

In addition, evidence has shown that stressors, especially the way of coping with a stressful situation, may interact with a variety of brain process, including the self-regulation process and cognitive control [14]. It is likely that the association between EF and effectiveness of PA interventions is conditional on the reaction to the work-related stress levels. The job demand-control (JDC) model has been frequently applied to evaluate work-related stress and the capability of coping with the stress [15,16]. According to the combinations of sub-items, the JDC model classifies four job stressor situations—high-strain, low-strain, passive, and active jobs [15,16]. The JDC model seems relevant to self-regulation and workplace-based intervention studies targeting PA-behavior, but it has been rarely investigated in previous studies. This is relevant to health promotion programs in a workplace-based setting because the JDC model reflects individual control levels over the work environmental factors.

In the current study, using data from a three-armed cluster randomized controlled trail in office workers [17], we examined the effect of individual EF difference on the effectiveness of interventions targeting PA behavior. Specifically, we aimed to investigate (1) the association between baseline EF and the intervention effect on changes of PA-behaviors; (2) whether and to what extent the influence of EF on the intervention effectiveness varies by JDC categories.

## 2. Materials and Methods

### 2.1. Design and Setting

This study was based on a single-blind, parallel-group, randomized trial with multi-component interventions conducted in Stockholm and Gothenburg. The recruitment of study participants started 15 March 2018 and was completed by 31 November 2018 (International Trial Registry no. ISRCTN92968402, registration date 27 February 2018). The hypothesis of this trial was that multi-component interventions of PA may show different effects on behavioral patterns, including levels of PA and sedentary time. The rationale, design, and detailed methods of this RCT study have been presented previously [17,18]. All participants answered surveys online and participated in device-measured PA behavior measurements at both pre- and post-intervention times. Data collection and analysis of primary outcomes and other measurements (e.g., cognitive tests) were carried out in the same manner before and after the intervention. The ethical approval was obtained from the Stockholm regional ethical review board (no. 2017/2409-31/1). We received the written informed consent forms from all participants before the data collection started.

### 2.2. Participants

The target population in this trial was office workers from two Swedish product- and service-producing companies (ICA-gruppen and Intrum) [17]. In collaboration with the two companies, we have established a long-term collaboration on the research theme of “PA and health in office workers”. The current study is a continuation of our previous project which is based on a cross-sectional design and to observe the association between PA and health status among the employees. During the recruitment process of current project, we first presented the aims of the project and research questions in the personnel meetings in each company, and recruitment advertisement was uploaded in the internal webpage of each company [17,18]. Invitation emails were then sent out to each employee, and they could sign up online for a willingness to participate. After this, we assessed the eligibility of each participant according to pre-defined selection criteria. Briefly, the eligibility criterions in recruiting study participants include (1) men and women aged 18–70 years; (2) who were with full-time working contract or status during the study period; (3) who were able to stand and exercise; and (4) their PA level that was measured by device-measured moderate-to-vigorous intensity PA (MVPA) ≤ 30 min/day in prolonged bouts (≥10 min) every day. We did not consider the level of sedentary behavior in our selection criterions because findings from a previous cross-sectional study observed that almost all office workers had a high level of sedentary behavior [17]. The reason for adding the third eligibility item was to exclude disabled persons or those with certain conditions that may hinder us to detect the intervention effectiveness. The recruitment process followed previous intervention studies in office workers [17,18], and the availability of office workers was reflected by the recruitment rate.

**Figure 1:**
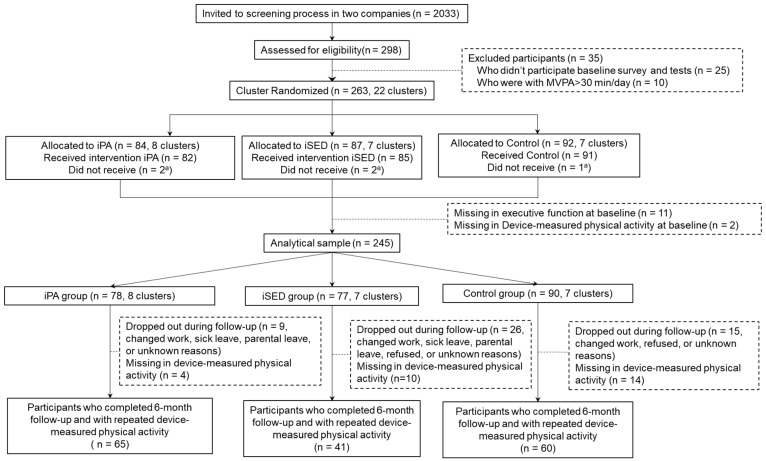
Flow chart of study participants in the multi-component intervention targeting physical activity and sedentary behavior. ^a^ Those who did not receive interventions were because they dropped out before notified of group allocation, or changed work during the intervention period.

In total, among 298 who agreed to participate our project, 263 were eligible. Finally, we recruited 245 participants in the current study (Figure 1). To avoid the effect of inconsistency and influential factors on the further analysis, the final analytical sample was obtained after the data clean and data organization procedure.

### 2.3. Randomization and Interventions

At each company, cluster randomization was applied to sample the study participants. Human resources officers at each company grouped office workers in the unit of teams (≥5 individuals per team). The essential criteria related to the selection of teams were: (1) Including a team/line manager; (2) having regular group meetings; and (3) having limited meetings with other teams [19]. The single-blinded (blinded outcome assessors) randomization was applied to allocate teams into two multi-component intervention groups and a control group [17]. Groups were randomly allocated (1:1:1) with stratification for company and cluster size (large vs. small). We applied matched randomization to realize logistical capacity. The random allocation sequence was set up using a computer-generated random number list. Each participant was followed until the last participant randomized had completed the 6-month visit.

The two multi-component interventions aimed to reduce sedentary behavior (iSED) or promote moderate to vigorous intensity PA (iPA). To ultimately benefit mental health and cognition, iSED arm aimed to reduce and break up prolonged sitting, while the iPA arm aimed to promote MVPA. The interventions focused on both work- and leisure-time, and were conducted through three levels: individual, environmental, and organizational. (1) At the individual level, cognitive behavioral therapy (CBT)-based face-to-face motivational counseling were conducted by a trained professional health coach in each arm. Participants in the iSED arm received CBT-based motivational counseling towards reducing time spent in sedentary behavior and breaking up prolonged sitting, as well as individual feedback on sedentary behavior. Participants in the iPA arm received the counseling towards increasing the time spent in MVPA, and feedbacks on the activity. In total, there were five sessions, three individual (45–60 min) and two group sessions (90 min), spread out during the 6-month intervention period (see details in Table 1) [19,20]. The content of the counselling sessions was standardized using manuals with checklists. (2) At the environmental level, within the iSED arm, meetings were implemented in a standing or walking format, which were initiated by team leaders. Within the iPA arm, exercise sessions and lunch walks were organized by the team leaders, while 6-month access to a commercial gym as well as company bikes were provided to the participant. (3) At the organizational level, the employees in the iSED arm were encouraged by the team leader to reduce sedentary behavior at work using their sit–stand desk or during meetings. Participants in the iPA arm were encouraged by the team leaders to be physically active at both work- and leisure-time, including communication between home and work. The wait-list passive control group were assigned to one of the intervention groups after 6 months.

Standardized processes were performed during the entire intervention period to ensure each team leader understood and implemented their roles in the interventions [17,18].

### 2.4. Data Collection and Measurements

Data collection of this trial varied by individuals. The pre-intervention data collection was conducted 7 days before the scheduled individual CBT-based face-to-face motivational counseling meeting. The start date of data collection for the first participant was 2 April 2018. We collected post-intervention data as soon as the individuals had completed the last CBT-based group intervention session (the last day of data collection: 31 May 2019). All participants responded to online surveys at baseline and 6-month follow-up, to collect information on demographic factors, health, work situation, work environment, and lifestyle habits [17]. Fitness level of participants was estimated via a submaximal fitness test performed on a cycle ergometer [17,21]. Specifically, during a sub maximal cycle ergometer test, we recorded the heart rates that response to different sub maximal rates of work—higher work rate and the standard work rate. Cardiovascular fitness, or maximal oxygen consumption, was estimated by sex-specific equations based on the difference in heart rates response between the two different sub maximal rates of work. It can be expressed as an absolute value (liter pre minute) or as a relative value (milliliters per minute per body mass). At baseline, age was reported by the participants as age at last birthday and was treated as a continuous variable in the analysis. Self-reported gender was dichotomized into female and male. We obtained the education background of each participant as the years of formal education completed.

### 2.5. Outcomes: Device-Measured PA and Sedentary Behavior

In this study, before and after the interventions, we obtained PA patterns and sedentary time using a GT3X accelerometer (Actigraph Inc. Pensacola, FL, USA). We instructed the participants to wear the GT3X for 7 days before the intervention and 7 days after the interventions—on the hip during wake time and move it to the left wrist during the in-bed time. While the sleep duration was recorded by the participants from the time that they began trying to fall asleep till the time they got out of bed in a daily diary [22,23]. Around 1% of participants at baseline and 3% of participants after intervention were with missing value in the diary information, we therefore applied a standard sleep time for those with missing value from 11 PM AM to 6 AM.

Tri-axial acceleration was used to measure the time spent in PA at different intensities. The inclusion criterion for accelerometer data was a minimum 600 min of valid wear time during waking hours on at least 4 days. According to the spectrum of acceleration from vector magnitude using accelerometer, PA was classified into five categories: sedentary time (<1.5 metabolic equivalents [METs]), light PA (LIPA, 1.5–3 METs), moderate PA (MPA, 3–6 METs), and vigorous-intensity PA (VPA, 6–8.9 METs), and very vigorous-intensity PA (VVPA, ≥9 METs). In the current study, we applied the percentage of wear time over an average of all available days on different physical activities as our outcomes.

### 2.6. Executive Function

We employed a comprehensive cognitive test battery (E-prime 2.0, psychology software Tools Inc, Sharpsburg, MD, USA) to test the cognitive function of study participants. At baseline, we engaged the paper and pen tests in Trail Making Test-B (TMT-B), and Stroop test, as well as computer-based test in n-back to evaluate the EF [17,24].

TMT-B neuropsychological test consists of circles that include both numbers (1–13) and letters (A–L), and the participants were requested to draw lines to connect the circles in an ascending pattern between the numbers and letters (i.e., 1-A-2-B-3-C). The participants were instructed to connect the circles as quickly as possible, without making mistakes or lifting the pen from the paper. TMT-B was scored as the number of seconds required to complete the test, and the lower score reveals better performance [25].

A modified Stroop test was applied to test the delay in reaction time between compatible and incompatible conditions. The participants were asked to answer as fast as possible the printed color of a written word, and the written word is describing color and it may be mismatched with the printed color (e.g., the word “red” printed in green ink instead of red ink). The Stroop test was scored as the number of seconds required to complete the test, and the lower score reveals better performance [21].

We engaged a computerized version of n-back task to the participants. The participants were presented with a sequence of stimuli (numbers), and asked to indicate with keyboard presses whether the current stimulus matches the one from two steps earlier in the sequence (e.g., the bold number from the following sequence of stimuli “1 3 1 7 9 8 9”). The n-back test was scored as 2-back accuracy and response time (second) on correct answers [21].

We first calculated reverse scores of TMT-B and Stroop by calculating the distance between the maximum value and individual score, and then standardized TMT-B reverse score, Stroop reverse score, and n-back accuracy score, respectively. The average score of the three standardized scores was finally calculated to indicate the EF, and a higher score reveals a better EF.

### 2.7. Assessments of JDC Model

The JDC model (Supplementary Figure S1) was developed by Robert Karasek in 1979 to define workload and job-related strain, and consists of psychosocial demands at work and job control [15]. We applied a Swedish version of the demand-control questionnaire (DCQ) to assess the JDC model [26,27,28,29]. Psychosocial demands at work were measured using five questions. (1) Do you have to work very fast? (2) Does your work demand too much effort? (3) Do you have to work very intensively? (4) Do you have enough time to do everything? (5) Does your work often involve conflicting demands? Job control was measured by six questions from DCQ and involves skill discretion and decision authority. (1) Does your work demand a high level of skill or expertise? (2) Does your work require ingenuity? (3) Do you have to do the same thing repeatedly? (4) Do you have the possibility of learning new things through your work? (5) Do you have a choice in deciding how to do your work? (6) Do you have a choice in deciding what you do at work [26]. These questions were quantified on a scale from 1 = “very rare” to 5 = “very often”. Except for answers to question 4 of the demands dimension and question 3 of the control dimension, the responses to the other nine questions were converted into 1 = “no, hardly ever/never” to 5 = “yes and frequently”. Response scores for job demands and job control were averaged, respectively. Higher score indicates higher demands or control. Based on the combination of job demands and control, four categories were calculated (Supplementary Figure S1): high-strain jobs (high demands, low control), low-strain jobs (low demands, high control), passive jobs (low demands, low control), or active jobs (high demands, high control).

### 2.8. Statistical Analysis

Baseline characteristics of the participants by intervention groups were presented with mean (standard deviation, SD) or frequency (%), and were compared by ANOVA for continuous variables with normal distribution and constant variance between groups, or by chi-square test for categorical variables. We first applied linear mixed-effects models to investigate whether EF may modify the intervention effect on PA pattern independently, i.e., changes in proportion of time spent on sleep, sedentary behavior, and various levels of PA. We further introduced the JDC categories and their interactions with EF in the models, to estimate the interactive effect between EF and JDC categories on the effectiveness of interventions. We estimated β-coefficients and 95% confidence interval (CI) of three-way interactions among intervention groups (control, iPA, and iSED groups), time, and modifiers (i.e., EF, JDC categories, and interactions between EF and JDC categories) in the linear mixed effect model. These β-coefficients (95% CI) were estimated as the fixed effect in the linear mixed effect model, considering the random intercept of individual’s differences at pre-intervention and random slope of individual’s changes between pre- and post-intervention. Covariates in the analysis include age, sex, education, clusters, and cardiovascular fitness. We performed fitness test of model by estimating the Akaike’s and Schwarz’s Bayesian information criteria, and the heteroscedasticity were tested by plotting the residuals with predictors. We conducted additional analyses by merging time spent on MPA, VPA, and VVPA into one sum—MVPA, and by relating EF and the JDC model to self-efficacy and perceived well-being.

## 3. Results

### 3.1. Characteristics of Study Participants

Of the 245 participants, 90 were in the control group, 78 were in the iPA group, and 77 were in the iSED group. Table 2 shows the detailed characteristics of the participants at baseline. The average age in the control group was significantly higher than the iPA group and iSED group. The number of years of education in the iPA group was significantly higher than that of the other groups. There was no significant difference in sex, fitness, job demand, job control, the JDC categories, EF, or time spent in PA-behaviors.

### 3.2. The Effect of JDC Categories on the Effectiveness of Interventions

**Figure 2:**
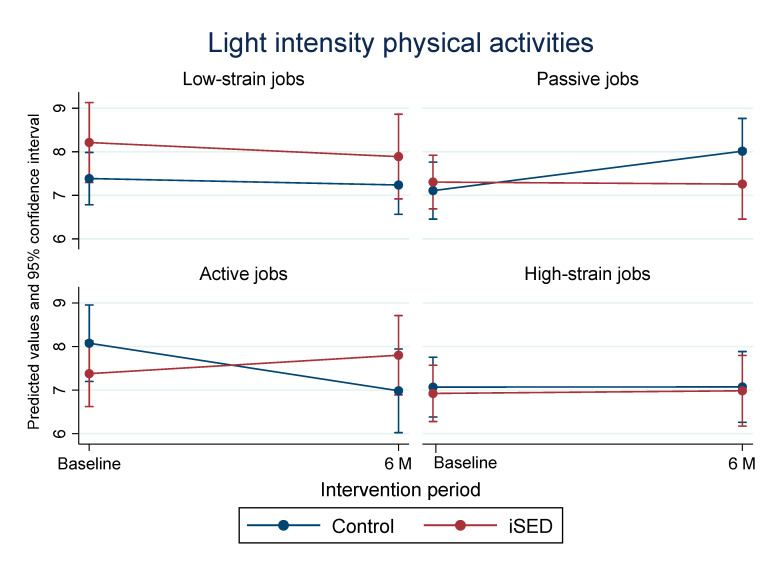
The association of the demand and control model with interventions and changes in light intensity physical activity.

When relating the JDC model to the intervention effects, results showed that active jobs were significantly associated with the intervention effect on LIPA, especially for the iSED group (β-coefficient for three-way interactions: 1.65, 95%CI [0.05, 3.46], *p* = 0.044). Figure 2 presents the changes in LIPA between the control group and the iSED group among the four JDC categories. Specifically, among participants with active jobs, LIPA was significantly increased in the iSED group in comparison to the control group (difference in slope: 1.69, 95%CI [0.15, 3.24], *p* = 0.032).

### 3.3. The Effect of EF on the Effectiveness of Interventions

Table 3 showed that compared to the control group, participants with higher EF in the iSED group presented a significant increase in sleep time (β-coefficient: 3.33, 95% CI [1.11, 5.55], *p* = 0.003) and a decrease in sedentary behavior (−2.76, 95% CI [−5.51, −0.01], *p* = 0.049).

### 3.4. The Joint Effect of EF and the JDC Categories on Effectiveness of Intervention

**Figure 3:**
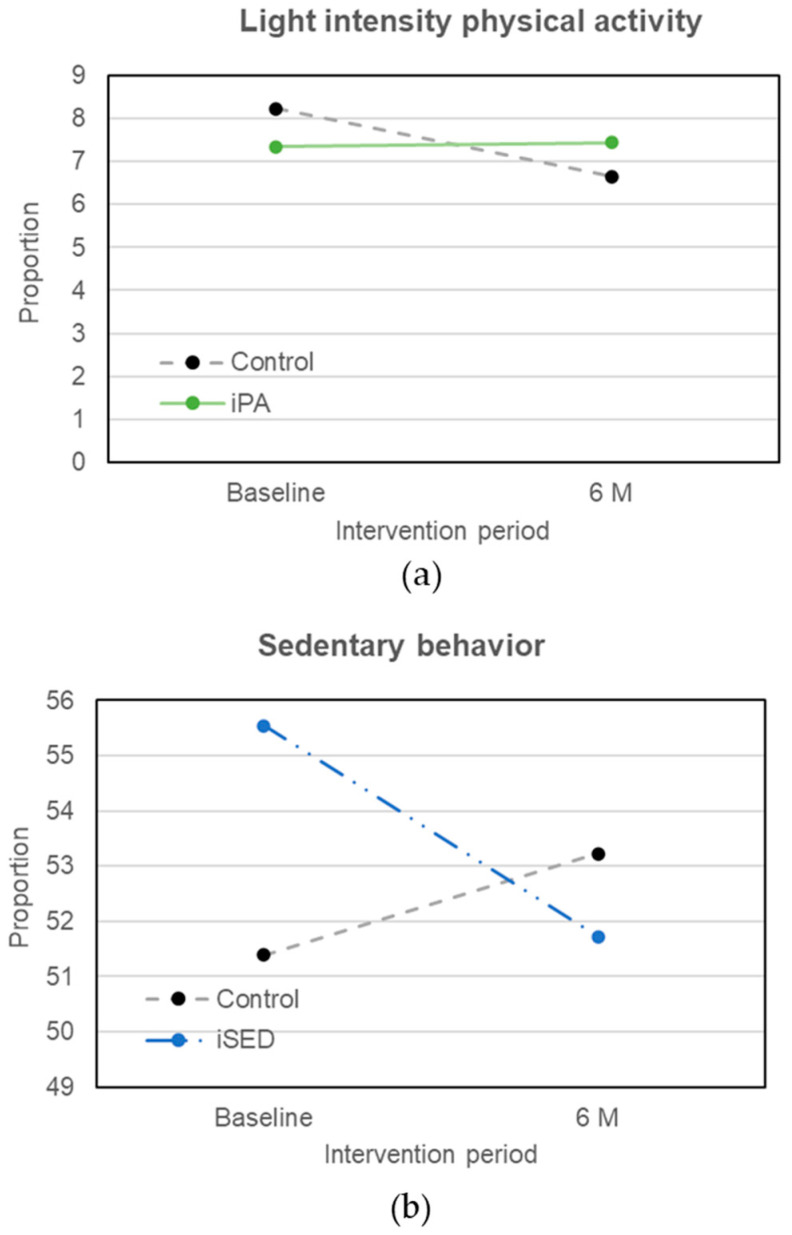
Intervention effect on physical activity patterns among those with a high executive function and active jobs. Notes: (**a**) shows changes in light intensity physical activity between control group and intervention group targeting physical activity; (**b**) shows the changes in sedentary time between control group and intervention group targeting sedentary behavior.

The high-strain jobs, defined as high job demands in combination with low job control, have been observed to associate with an inactive lifestyle, as well as increased risk of cognitive deficits and cardiovascular events [19,20,26,30,31]. Active jobs (high job demands and high job control), in contrast, may affect job characteristics via adaptive or maladaptive self-regulation strategies, and lead to a healthy and active lifestyle [14]. To further study whether the effect of EF on effectiveness of intervention varies by the JDC categories, we generated a joint variable between EF and JDC categories with four categories: (1) low EF, non-active jobs; (2) high EF, non-active jobs; (3) low EF, active jobs; (4) high EF, active jobs. The results showed that among participants who had both high EF and active jobs, compared to the control group, iPA group showed a substantial increase in LIPA level (β-coefficient,1.58 95% CI [0.10, 3.05], *p* = 0.036, Figure 3a), and iSED group showed a tendency of reduced sedentary behavior (β-coefficient, −5.35, 95% CI [−10.80, 0.10], *p* = 0.054, Figure 3b).

### 3.5. Additional Analysis

Results demonstrated that neither EF nor four job stressor situations were associated with the intervention effect on the change of MVPA. There was no joint effect of active jobs and high EF on the intervention effect on changes in self-efficacy or well-being.

## 4. Discussion

### 4.1. Main Findings

Based on the three-arm intervention study targeting PA and sedentary behavior, the findings are summarized as follows. (1) A higher EF was associated with the intervention effect on increased sleep time and reduced sedentary time in the iSED group. (2) Participants with active jobs presented significantly increased LIPA in the iSED group in comparison to the control group. (3) There was a joint effect of EF and the JDC categories on the intervention effect—among participants with high EF and active jobs, compared to the control group, iPA group showed a substantial increase in LIPA, and iSED group showed a tendency of sedentary behavior reduction.

### 4.2. Comparison with Previous Studies

This is the first study to investigate the self-regulation related factors (EF and the JDC categories) on the effectiveness of PA interventions in office workers. Most of previous research focused on the effect of PA interventions on EF [3,32,33,34]. Although the effect of PA on EF has been acknowledged in population-based studies [35], intervention studies targeting promoting PA or reducing sedentary behavior have generated inconsistent results on the effect of PA on EF in adulthood or employed populations [34]. A few studies showed that higher EF was associated with greater adherence to exercise or PA engagement, but the findings were mostly from the population-based design involving older adults [3,36]. A growing number of studies support the link between the JDC model and PA level in the employed populations [37,38,39]. A systematic review has concluded that high-strain jobs reduce the efforts to be physically active [37]. Findings from a large-scale study of adults further proved the hypothesis that greater control at work was associated with more PA in both females and males [38]. Using sensor-based PA data, a cross-sectional study involving a workplace-based population found that a high job demand was associated with a low MVPA and VPA [39]. Findings from the current study have extended the previous findings by investigating behavioral changes in PA-related interventions in workplace-based settings in relation to EF, the JDC model, and their interactions.

### 4.3. Possible Explanations and Mechanisms

The findings from the current study indicate an interactive effect of EF and work stressors on PA behavioral changes in the workplace-based setting. It has been suggested that individual differences in EF may explain the variance of health behavior performance with short periods of time (e.g., 7 days) or long term (e.g., months or years) [4,5,40]. Self-regulation theory has suggested that EF may affect the efficacy of CBT, such as the ability to organize and plan activities, shift and direct attention, as well as adjust behavior according to long-term goals [2,3]. One interpretation of the JDC model is that the imbalance of JDC can lead to diverse health outcomes via influencing healthy behaviors [41]. For example, the association between high job demands and good health only exists in those with a high level of job control [41]. Thus, employees with a high job control may present more flexibility in engaging in PA at both work- and leisure-time. Previous studies have shown that compared to individuals with non-active jobs, those with active jobs tend to have a higher level of leisure time activity, more positive learning tendency, and more psychological de-mands [42]. In addition, a low level of job control has been shown to prospectively increase the risk of low PA [31]. It is possible that PA is one of the stress modifiers that might help individuals against work-related stress. Therefore, compared to other employees with non-active jobs, those with a high level of EF and active jobs may benefit most from the CBT-based motivation counseling in each intervention arm, and therefore presented the most significant intervention results on PA behavioral changes.

### 4.4. Strength and Limitations

This study has several strengths. We applied sensor-based measurements of PA level and sedentary time. Using linear mixed-effects models allowed us to investigate the between individual changes after considering within individual differences in a longitudinal study design. Multi-arm design that distinguishes PA and sedentary behaviors made it possible to evaluate the intervention effect towards specific domains in health behaviors. However, several limitations remain in this study. (1) Although the sample size is sufficient to conduct a statistical inference after controlling for study design [17], the sample size may limit our ability to detect the true significant results, e.g., the association of job demands and job control with the intervention effect. (2) Sleep time in the current study was assessed by the time in bed at night. Future studies are encouraged to apply device-based measures of sleep duration and sleep cycle. This will provide more insight into the association between daytime activities and sleep. (3) Similar to other intervention studies, potential selection bias may exist in our study. Excluding participants with a high level of PA may lead to the study population not being representative of the target population. (4) Measurement bias may occur when the self-reported information was applied, e.g., DCQ. Future studies are recommended to employ reliability measurement in collecting questionnaire data, such as measurements for inter-rater reliability or test–retest reliability.

## 5. Conclusions

In light of our results, individual difference in EF seems to affect the effectiveness of intervention targeting PA-related behaviors in office workers. In addition, office workers with higher level of EF and better capability of coping with work-related stress showed considerably greater intervention effectiveness on PA and sedentary outcomes. This study extended understanding of the bidirection between EF and healthy behaviors, and acknowledged that cognitive control and self-reaction to stressors may adjust the effectiveness of health promotion interventions. It is necessary to consider the self-regulation related factors in designing an intervention study targeting healthy behaviors. Future studies are encouraged to evaluate whether and to what extent variations in behavioral intervention success can be explained by self-regulation theory and to verify the influential effect of other factors on the intervention effectiveness, e.g., social-economic status.

Since our study revealed that certain characteristics of office workers (i.e., high EF and active job) may benefit most from a health promotion program or PA-related intervention, future health promotion programs should place special attention on other sub-groups, e.g., the group who are with lower EF and less capable of coping with stressors.

## Figures and Tables

**Table 1:** Motivational counselling sessions during intervention process.

	Duration	Performance	Break Period
Session 1	60 min	Face-to-face individual session	1 week after baseline data collection
Session 2	45 min	Face-to-face individual session	1–2 weeks after session 1
Session 3	90 min	Receiving individual feedbacks Group session	6–9 weeks after session 1
Session 4	45 min	Face-to-face individual session	13–14 weeks after session 1
Session 5	90 min	Group session	23 weeks after session 1

**Table 2:** Characteristics of participants at baseline (*n* = 245).

	Control Group (*n* = 90)	iPA Group (*n* = 78)	iSED Group (*n* = 77)	*p*-Value
Age (year), Mean (SD)	44.27 (7.76)	40.69 (8.80)	40.39 (8.72)	0.004
Female, *n* (%)	69 (78.67)	61 (78.21)	49 (63.64)	0.077
Education (year), Mean (SD) ^a^	14.81 (2.03)	15.29 (2.06)	14.45 (1.94)	0.037
Fitness level (mL.kg^−1^.min^−1^), Mean (SD)	36.17 (7.82)	36.82 (7.23)	38.90 (7.39)	0.055
Job demands, mean (SD) ^a^				
Low	52 (59.09)	34 (43.59)	35 (47.95)	
High	36 (40.91)	44 (56.41)	38 (52.05)	0.118
Job control, mean (SD) ^a^				
Low	43 (52.44)	42 (55.26)	47 (62.67)	
High	39 (47.56)	34 (44.74)	28 (37.33)	0.415
Job Demands–Control categories, *n* (%)				
Low-strain jobs	26 (32.10)	16 (21.05)	11 (14.86)	
Passive-strain jobs	22 (27.16)	17 (22.37)	25 (33.78)	
Active jobs	12 (14.81)	18 (23.68)	16 (21.62)	
High-strain jobs	21 (25.93)	25 (32.89)	22 (29.73)	0.156
Executive function, Mean (SD)	−0.04 (0.87)	0.00 (0.63)	0.07 (0.73)	0.644
Proportion of time spent on physical activity behaviors				
Sleep (%)	32.02 (2.84)	31.86 (2.70)	31.43 (3.25)	0.418
Sedentary time (%)	53.11 (3.81)	53.18 (3.76)	53.85 (3.77)	0.390
LIPA (%)	7.44 (1.72)	7.43 (1.65)	7.38 (1.66)	0.972
MPA (%)	6.72 (1.66)	6.81 (1.63)	6.65 (1.78)	0.75
VPA (%)	0.55 (0.49)	0.54 (0.43)	0.51 (0.39)	0.866
VVPA (%)	0.17 (0.16)	0.18 (0.16)	0.17 (0.12)	0.7433

Notes. SD = Standard Deviation, iPA = Increasing physical activity, iSED = intervention to reduce sedentary behavior, LIPA = light physical activity, MPA = moderate physical activity, VPA = vigorous physical activity, VVPA = very vigorous physical activity. ^a^ There are 6 missing values for education level, 5 for job demands, 14 for job control, 8 for social support.

**Table 3:** The association of executive function with changes in physical activity pattern before and after intervention.

	Sleep %		Sedentary Time %		LIPA %		MPA %		VPA %		VVPA %	
	β-coefficient (95% CI)	*p*-value	β-coefficient (95% CI)	*p*-value	β-coefficient (95% CI)	*p*-value	β-coefficient (95% CI)	*p*-value	β-coefficient (95% CI)	*p*-value	β-coefficient (95% CI)	*p*-value
**EF Function × Time × Int**												
***EF, z-score***												
iPA × Time × EF	0.35 (−0.96, 1.65)	0.594	−0.30 (−1.90, 1.31)	0.718	0.03 (−0.65, 0.71)	0.937	−0.02 (−0.71, 0.68)	0.961	−0.05 (−0.23, 0.13)	0.606	−0.02 (−0.09, 0.05)	0.614
iSED × Time × EF	1.52 (−0.01, 3.06)	0.052	−1.61 (−3.50, 0.28)	0.095	0.04 (−0.77, 0.85)	0.929	−0.17 (−0.99, 0.65)	0.686	0.19 (−0.02, 0.40)	0.083	0.01 (−0.07, 0.09)	0.883
***Binary of EF***												
iPA × Time × EF Top median	1.09 (−0.82, 3.00)	0.264	−0.16 (−2.53, 2.21)	0.894	−0.49 (−1.49, 0.52)	0.342	−0.40 (−1.43, 0.62)	0.442	−0.03 (−0.29, 0.24)	0.834	−0.01 (−0.11, 0.09)	0.838
iSED × Time × EF Top median	3.33 (1.11, 5.55)	0.003	−2.76 (−5.51, −0.01)	0.049	−0.54 (−1.71, 0.62)	0.362	−0.28 (−1.47, 0.92)	0.650	0.25 (−0.06, 0.56)	0.119	−0.00 (−0.12, 0.12)	0.981

Notes. Findings were derived from the linear mixed models adjusting for age, gender, education, clusters, and cardiovascular fitness level. EF = Executive function, Int = Intervention groups, iPA = Increasing physical activity, iSED = intervention to reduce sedentary behavior, LIPA = light physical activity, MPA = moderate physical activity, VPA = vigorous physical activity, VVPA = very vigorous physical activity.

## Data Availability

The data presented in this study are available on request.

## Supplementary Materials

The following are available online at https://www.mdpi.com/article/10.3390/ijerph19010266/s1, Figure S1: Job Demand Control Model by Robert Karasek.

## References

[1] Bull F.C., Al-Ansari S.S., Biddle S., Borodulin K., Buman M.P., Cardon G., Carty C., Chaput J.-P., Chastin S., Chou R. (2020). World Health Organization 2020 Guidelines on Physical Activity and Sedentary Behaviour. Br. J. Sports Med..

[2] Buckley J., Cohen J.D., Kramer A.F., McAuley E., Mullen S.P. (2014). Cognitive control in the self-regulation of physical activity and sedentary behavior. Front. Hum. Neurosci..

[3] McAuley E., Mullen S.P., Szabo A.N., White S.M., Wójcicki T.R., Mailey E.L., Gothe N.P., Olson E.A., Voss M., Erickson K. (2011). Self-regulatory processes and exercise adherence in older adults: Executive function and self-efficacy effects. Am. J. Prev. Med..

[4] Daly M., McMinn D., Allan J.L. (2015). A Bidirectional Relationship between Physical Activity and Executive Function in Older Adults. Front. Hum. Neurosci..

[5] Allan J.L., McMinn D., Daly M. (2016). A Bidirectional Relationship between Executive Function and Health Behavior: Evidence, Implications, and Future Directions. Front. Neurosci..

[6] Merz E.C., Landry S.H., Montroy J.J., Williams J.M. (2017). Bidirectional Associations Between Parental Responsiveness and Executive Function During Early Childhood. Soc. Dev..

[7] Danquah I.H., Kloster S., Holtermann A., Aadahl M., Bauman A., Ersbøll A.K., Tolstrup J.S. (2017). Take a Stand!—A multi-component intervention aimed at reducing sitting time among office workers—A cluster randomized trial. Int. J. Epidemiol..

[8] Aittasalo M., Miilunpalo S., Suni J. (2004). The Effectiveness of Physical Activity Counseling in a Work-Site Setting: A Randomized, Controlled Trial. Patient Educ. Couns..

[9] Edwardson C.L., Yates T., Biddle S.J.H., Davies M.J., Dunstan D.W., Esliger D.W., Gray L.J., Jackson B., O’Connell S.E., Waheed G. (2018). Effectiveness of the Stand More at (SMArT) Work Intervention: Cluster Randomised Controlled Trial. BMJ.

[10] Edmunds S., Stephenson D., Clow A. (2013). The Effects of a Physical Activity Intervention on Employees in Small and Medium Enterprises: A Mixed Methods Study. Work.

[11] Healy G.N., Eakin E.G., Lamontagne A.D., Owen N., Winkler E.A., Wiesner G., Gunning L., Neuhaus M., Lawler S., Fjeldsoe B.S. (2013). Reducing sitting time in office workers: Short-term efficacy of a multicomponent intervention. Prev. Med..

[12] Urda J.L., Lynn J.S., Gorman A., Larouere B. (2016). Effects of a Minimal Workplace Intervention to Reduce Sedentary Behaviors and Improve Perceived Wellness in Middle-Aged Women Office Workers. J. Phys. Act. Health.

[13] Peterman J.E., Healy G.N., Winkler E.A., Moodie M., Eakin E.G., Lawler S.P., Owen N., Dunstan D.W., LaMontagne A.D. (2019). A cluster randomized controlled trial to reduce office workers’ sitting time: Effect on productivity outcomes. Scand. J. Work Environ. Health.

[14] Bakker A.B., de Vries J.D. (2021). Job Demands—Resources Theory and Self-Regulation: New Explanations and Remedies for Job Burnout. Anxiety Stress Coping.

[15] Karasek R.A. (1979). Job Demands, Job Decision Latitude, and Mental Strain: Implications for Job Redesign. Adm. Sci. Q..

[16] Theorell T., Karasek R.A. (1996). Current Issues Relating to Psychosocial Job Strain and Cardiovascular Disease Research. J. Occup. Health Psychol..

[17] Nooijen C.F.J., Blom V., Ekblom Ö., Ekblom M.M., Kallings L.V. (2019). Improving Office Workers’ Mental Health and Cognition: A 3—Arm Cluster Randomized Controlled Trial Targeting Physical Activity and Sedentary Behavior in Multi—Component Inter-ventions. BMC Public Health.

[18] Nooijen C.F.J., Blom V., Ekblom Ö., Heiland E.G., Larisch L.-M., Bojsen-Møller E., Ekblom M.M., Kallings L.V. (2020). The Effectiveness of Multi-Component Interventions Targeting Physical Activity or Sedentary Behaviour amongst Office Workers: A Three—Arm Cluster Randomised Controlled Trial. BMC Public Health.

[19] Kivimäki M., Nyberg S.T., Batty G.D., Fransson E.I., Heikkilä K., Alfredsson L., Bjorner J.B., Borritz M., Burr H., Casini A. (2012). Job Strain as a Risk Factor for Coronary Heart Disease: A Collaborative Meta-Analysis of Individual Participant Data. Lancet.

[20] Elovainio M., Feme J.E., Singh-Manoux A., Gimeno D., De Vogli R., Shipley M.J., Vahtera J., Brunner E.J., Marmot M.G., Kivimäki M. (2009). Cumulative Exposure to High-Strain and Active Jobs as Predictors of Cognitive Function: The Whitehall II Study. Occup. Environ. Med..

[21] Bojsen-Møller E., Boraxbekk C.J., Ekblom Ö., Blom V., Ekblom M.M. (2019). Relationships between Physical Activity, Sedentary Behaviour and Cognitive Functions in Office Workers. Int. J. Environ. Res. Public Health.

[22] Fridolfsson J., Börjesson M., Buck C., Ekblom Ö., Ekblom-Bak E., Hunsberger M., Lissner L., Arvidsson D. (2019). Effects of Fre-quency Filtering on Intensity and Noise in Accelerometer-Based Physical Activity Measurements. Sensors.

[23] Larisch L.-M., Bojsen-Møller E., Nooijen C.F.J., Blom V., Ekblom M., Ekblom Ö., Arvidsson D., Fridolfsson J., Hallman D.M., Mathiassen S.E. (2021). Effects of Two Randomized and Controlled Multi-Component Interventions Focusing On 24-Hour Movement Behavior among Office Workers: A Compositional Data Analysis. Int. J. Environ. Res. Public Health.

[24] Nooijen C.F.J., Kallings L.V., Blom V., Ekblom Ö., Forsell Y., Ekblom M.M. (2018). Common Perceived Barriers and Facilitators for Reducing Sedentary Behaviour among Office Workers. Int. J. Environ. Res. Public Health.

[25] Tombaugh T.N. (2004). Trail Making Test A and B: Normative data stratified by age and education. Arch. Clin. Neuropsychol..

[26] Tan E.C., Pan K.-Y., Magnusson Hanson L.L., Fastbom J., Westerlund H., Wang H.X. (2020). Psychosocial Job Strain and Polypharmacy: A National Cohort Study. Scand. J. Work, Environ. Health.

[27] Pelfrene E., Vlerick P., Mak R.P., De Smet P., Kornitzer M., De Backer G. (2001). Scale Reliability and Validity of the Karasek’ Job Demand-Control-Support Model in the Belstress Study. Work Stress.

[28] Fransson E.I., Nyberg S.T., Heikkilä K., Alfredsson L., Bacquer D.D., Batty G.D., Bonenfant S., Casini A., Clays E., Goldberg M. (2012). Comparison of Alternative Versions of the Job Demand-Control Scales in 17 European Cohort Studies: The IPD-Work Consortium. BMC Public Health.

[29] Chungkham H.S., Ingre M., Karasek R., Westerlund H., Theorell T. (2013). Factor Structure and Longitudinal Measurement In-variance of the Demand Control Support Model: An Evidence from the Swedish Longitudinal Occupational Survey of Health (SLOSH). PLoS ONE.

[30] Nyberg S.T., Fransson E.I., Heikkilä K., Alfredsson L., Casini A., Clays E., De Bacquer D., Dragano N., Erbel R., Ferrie J.E. (2013). Job strain and cardiovascular disease risk factors: Meta-analysis of individual-participant data from 47,000 men and women. PLoS ONE.

[31] Fransson E.I., Heikkilä K., Nyberg S.T., Zins M., Westerlund H., Westerholm P., Väänänen A., Virtanen M., Vahtera J., Theorell T. (2012). Job Strain as a Risk Factor for Leisure-Time Physical Inactivity: An Individual-Participant Meta-Analysis of up to 170,000 Men and Women. Am. J. Epidemiol..

[32] Álvarez-Bueno C., Pesce C., Cavero-Redondo I., Sánchez-López M., Martínez-Hortelano J.A., Martínez-Vizcaíno V. (2017). The Effect of Physical Activity Interventions on Children’s Cognition and Metacognition: A Systematic Review and Meta-Analysis. J. Am. Acad. Child Adolesc. Psychiatry.

[33] Brasure M., Desai P., Davila H., Nelson V.A., Calvert C., Jutkowitz E., Butler M., Fink H.A., Ratner E., Hemmy L.S. (2018). Physical Activity Interventions in Preventing Cognitive Decline and Alzheimer-Type Dementia a Systematic Review. Ann. Intern. Med..

[34] Erickson K.I., Hillman C., Stillman C.M., Ballard R.M., Bloodgood B., Conroy D.E., Macko R., Marquez D.X., Petruzzello S.J., Powell K.E. (2019). Physical Activity, Cognition, and Brain Outcomes: A Review of the 2018 Physical Activity Guidelines. Med. Sci. Sports Exerc..

[35] Ratey J.J., Loehr J.E. (2011). The Positive Impact of Physical Activity on Cognition during Adulthood: A Review of Underlying Mechanisms, Evidence and Recommendations. Rev. Neurosci..

[36] Eggermont L.H.P., Milberg W.P., Lipsitz L.A., Scherder E.J.A., Leveille S.G. (2009). Physical Activity and Executive Function in Aging: The MOBILIZE Boston Study. J. Am. Geriatr. Soc..

[37] Stults-Kolehmainen M.A., Sinha R. (2014). The Effects of Stress on Physical Activity and Exercise. Sports Med..

[38] Griep R.H., Nobre A.A., Alves M.G.D.M., Da Fonseca M.D.J.M., Cardoso L.D.O., Giatti L., Melo E.C.P., Toivanen S., Chor D. (2015). Job Strain and Unhealthy Lifestyle: Results from the Baseline Cohort Study, Brazilian Longitudinal Study of Adult Health (ELSA-Brasil). BMC Public Health.

[39] Larsson K., Ekblom Ö., Kallings L.V., Ekblom M., Blom V. (2019). Job Demand-Control-Support Model as Related to Objectively Measured Physical Activity and Sedentary Time in Working Women and Men. Int. J. Environ. Res. Public Health.

[40] Hall P.A., Fong G.T., Epp L.J., Elias L.J. (2008). Executive Function Moderates the Intention-Behavior Link for Physical Activity and Dietary Behavior. Psychol. Health.

[41] Gonzalez-Mulé E., Cockburn B.S. (2021). This Job Is (Literally) Killing Me: A Moderated-Mediated Model Linking Work Characteristics to Mortality. J. Appl. Psychol..

[42] Hellerstedt W.L., Jeffery R.W. (1997). The Association of Job Strain and Health Behaviours in Men and Women. Int. J. Epidemiol..

