# Supplementary figures and images for: The Role of Executive Function in the Effectiveness of Multi-Component Interventions Targeting Physical Activity Behavior in Office Workers
Source: Int J Environ Res Public Health. 2021 Dec 27;19(1):266. doi: 10.3390/ijerph19010266 (PMC8751160; doi:10.3390/ijerph19010266)

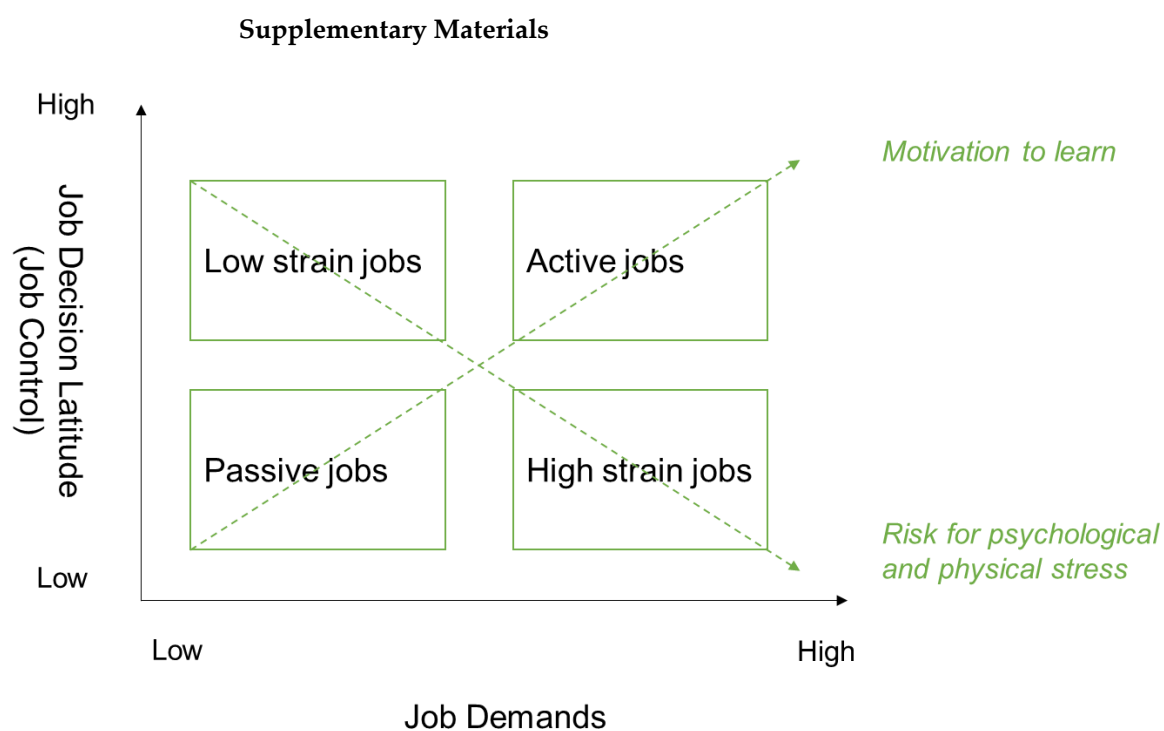

**Figure S1.** Job Demand Control Model by Robert Karasek.

Supplement: Supplementary file 1 [file ijerph-19-00266-s001.zip › ijerph-1466729-Supplementary.pdf]
